# Preliminary Study on Sensor-Based Detection of an Adherent Cell’s Pre-Detachment Moment in a MPWM Microfluidic Extraction System

**DOI:** 10.3390/s25092726

**Published:** 2025-04-25

**Authors:** Marius-Alexandru Dinca, Mihaita Nicolae Ardeleanu, Dan Constantin Puchianu, Gabriel Predusca

**Affiliations:** 1 Independent Researcher, 130004 Targoviste, Romania; alex.mdinca@gmail.com; 2 Faculty of Materials Engineering and Mechanics, Valahia University of Targoviste, 13 Aleea Sinaia Street, 130004 Targoviste, Romania; 3 Faculty of Electrical Engineering, Electronics and Information Technology, Valahia University of Targoviste, 13 Aleea Sinaia Street, 130004 Targoviste, Romania; dan.puchianu@valahia.ro (D.C.P.); gabriel.predusca@valahia.ro (G.P.)

**Keywords:** CCD sensor, adherent cell, cell pre-detachment moment detection, image processing, deep learning, microfluidics, micromechatronics

## Abstract

The extraction of adherent cells, such as B16 murine melanoma cells, from Petri dish cultures is critical in biomedical applications, including cell reprogramming, transplantation, and regenerative medicine. Traditional detachment methods—enzymatic, mechanical, or chemical—often compromise cell viability by altering membrane integrity and disrupting adhesion proteins. To address these challenges, this study investigated sensor-based detection of the pre-detachment phase in a MPWM (Microfluidic Pulse Width Modulation) extraction system. Our approach integrates a micromechatronic system with a microfluidic suction circuit, real-time CCD imaging, and computational analysis to detect and characterize the pre-detachment moment before full extraction. A precisely controlled hydrodynamic force field progressively disrupts adhesion in multiple stages, reducing mechanical stress and preserving cell integrity. Real-time video analysis enables continuous monitoring of positional dynamics and oscillatory responses. Image processing and deep learning algorithms determine object center coordinates, allowing the MPWM system to dynamically adjust suction parameters. This optimizes detachment while minimizing liquid absorption and reflux volume, ensuring efficient extraction. By combining microfluidics, sensor detection, and AI-driven image processing, this study established a non-invasive method for optimizing adherent cell detachment. These findings have significant implications for single-cell research, regenerative medicine, and high-throughput biotechnology, ensuring maximal viability and minimal perturbation.

## 1. Introduction

Adherent cell cultures, such as B16 murine melanoma cells, are widely used in biomedical research due to their ability to replicate in vivo cellular behavior under controlled laboratory conditions. The detachment and extraction of these cells from culture vessels are critical steps in various biotechnological and medical applications, including cell reprogramming, transplantation, regenerative medicine, and high-throughput drug screening. Traditional detachment techniques—such as enzymatic dissociation (e.g., trypsinization), mechanical scraping, or chemical dissociation—frequently compromise cell viability by disrupting cell membrane integrity and affecting cellular adhesion proteins, leading to unwanted stress and possible changes in cellular function [1].

To overcome these limitations, this study introduced a micromechatronic system that employs a MPWM (Microfluidic Pulse Width Modulation) microfluidic extraction circuit to facilitate a gentle and highly controlled cell detachment process. Microfluidic platforms have been shown to provide controlled environments for cell cultures, allowing precise regulation of microenvironmental factors [2]. The system generates a precisely regulated hydrodynamic force field, which progressively disrupts cell–substrate adhesion in multiple intermediate phases, minimizing mechanical stress and preserving biological integrity throughout the extraction process [3].

A key feature of this system is its ability to detect the pre-detachment phase of the adherent cell, a critical moment in the extraction process that occurs before the complete loss of adhesion. This detection is achieved through real-time video analysis, in which a CCD sensor continuously monitors the cell’s behavior. The recorded positional dynamics and oscillatory response provide insight into the cell’s mechanical resistance, allowing for adaptive control of suction parameters [4]. The system automatically adjusts pressure and suction duration, optimizing the extraction process while minimizing the volume of liquid absorbed alongside the cell. This, in turn, reduces the reflux liquid required for the final expulsion of the cell from the distal end of the extraction tube, ensuring an efficient and precise extraction mechanism. Additionally, image processing and deep learning algorithms are employed to track both the target cell’s position and the behavior of surrounding cells, ensuring precise extraction without unintentionally disrupting neighboring adherent cells. By integrating micromechatronics, microfluidics, sensor-based detection, real-time imaging, and artificial intelligence, this system establishes a high-precision, non-invasive approach to adherent cell extraction [5].

This preliminary study provides fundamental insights into sensor-assisted monitoring of the pre-detachment phase, with significant implications for single-cell research, regenerative medicine, and automated high-throughput biotechnological applications. By capturing and analyzing the early mechanical signals of detachment, the proposed system enables enhanced automation, precision, and reproducibility in cell extraction, minimizing biological perturbations and ensuring optimal cell viability [6].

Considering these advances, the current study proposes a novel sensor-based microfluidic system for detecting the pre-detachment moment in adherent cells. By combining micromechatronics, adaptive fluid control, CCD-based real-time monitoring, and AI-powered image analysis, the system aims to minimize stress during detachment and maximize post-extraction cell viability.

## 2. Related Works

The development of microfluidic systems for adherent cell extraction has been an important area of research, with significant advancements in sensor-based detection, microfluidic transport control, and precision manipulation at the picoliter scale. A key aspect of these systems is the integration of Microfluidic Pulse Width Modulation (MPWM) technology, which enables precise hydrodynamic force application for non-invasive cellular manipulation.

Advanced microfluidic systems have significantly enhanced control over cellular environments, enabling more precise and less invasive manipulation of adherent cells in biomedical research. Traditional methods of cell detachment—enzymatic, mechanical, or chemical—often disrupt the cell membrane and alter cellular function. Consequently, researchers have focused on developing microfluidic and sensor-based alternatives that offer higher precision and preserve cell viability.

Microfluidic perfusion systems provide stable and uniform culture conditions, allowing long-term real-time monitoring of biological cells with minimal disturbance to their physiological state [7]. These platforms mimic in vivo environments and ensure consistent nutrient flow and waste removal, making them ideal for sensitive applications such as regenerative medicine and single-cell analysis. Biosensors integrated into microfluidic systems offer valuable tools for assessing cell adhesion and detachment in real time. For instance, acoustic sensors can non-invasively detect viscoelastic changes at the cell–surface interface, revealing early signs of detachment or cellular stress [8]. These sensors contribute to the automation of the detachment process by providing continuous feedback on cell behavior.

In addition, recent advancements in sensor technologies allow for precise monitoring of physical parameters and metabolic activity within cell cultures. These sensors enhance control over factors such as pH, temperature, and metabolite concentration, which are critical for maintaining optimal cell function [9]. The integration of such sensors with intelligent control systems opens new opportunities for adaptive microfluidic detachment strategies. On a cellular level, precise intracellular delivery systems—using microfluidic or nanofluidic structures—have demonstrated the ability to manipulate cells without compromising their viability [10]. These systems, often guided by AI-based image processing, enable fine control of force application during detachment, further reducing the risk of cell damage.

Furthermore, microfluidic devices that allow real-time tracking and selective trapping of single cells have proven effective in isolating target cells for analysis or therapeutic purposes [11]. Such systems support dynamic analysis of cellular behavior in response to mechanical stimuli, offering critical insights into pre-detachment behavior.

Finally, optical biosensor chips that monitor both cell morphology and adhesion strength have shown great promise in distinguishing between healthy and diseased cells in situ. These systems provide a dual perspective—visual and mechanical—on the detachment process, thus contributing to enhanced precision in extraction protocols [12].

The concept of Microfluidic Pulse Width Modulation (MPWM) has been introduced as a high-precision method for controlling microfluidic transport at the nano-, pico-, and femtoliter scale. Ardeleanu et al. [13] proposed a PDMS-based sensor system capable of real-time measurement of MPWM-generated fluid volumes, using a circular-section microchannel fabricated via microwire molding techniques. Their study demonstrated that MPWM-driven microfluidic transport can be precisely modulated by tuning valve actuation times, thus allowing for the controlled displacement of minute liquid volumes. This approach has proven essential for automated microinjection and cell manipulation, highlighting the potential of MPWM for applications in biomedical research and single-cell studies.

The use of sensor-based microfluidic systems has been widely explored for biological sample handling and analysis. Traditional methods for fluid volume detection in microfluidic environments often rely on optical microscopy and image processing algorithms. Ardeleanu et al. [13] developed an optical volumetric measurement technique, leveraging real-time tracking of fluid boundaries within PDMS microchannels. This technique employs Digital Tracking Coordinate Systems (DTCS) to achieve submicron precision in fluid displacement measurement, which is critical for quantifying cell extraction forces and monitoring cell detachment dynamics.

In addition, various microfluidic sensors have been designed to monitor the behavior of biological entities within controlled environments. For instance, lab-on-a-chip (LOC) technologies have been utilized for biomolecular analysis, single-cell detection, and high-throughput screening [14]. These systems often integrate bioMEMS (biological microelectromechanical systems), allowing for real-time monitoring of microfluidic transport processes. The integration of MPWM-controlled microfluidics with high-resolution optical detection represents a significant advancement in non-invasive cellular manipulation techniques. The extraction of adherent cells from Petri dish cultures requires a high degree of precision in force application to avoid cell membrane rupture and ensure high viability post-extraction. Studies on hydrodynamic cell detachment mechanisms have explored the role of fluid shear stress in disrupting cell–substrate adhesion [15]. In the context of MPWM-driven systems, the ability to fine-tune suction force parameters based on real-time sensor feedback allows for controlled pre-detachment monitoring.

Recent advances in computational fluid dynamics (CFD) modeling have provided insights into the interaction between microfluidic flow patterns and adherent cell detachment dynamics. These models suggest that hydrodynamic force fields generated by pulse-modulated microfluidic circuits can be optimized to achieve gradual cell detachment, reducing mechanical stress and preserving cell morphology [16].

The development of advanced microfluidic and sensor-integrated platforms has driven considerable progress in the precise extraction and monitoring of adherent cells. Multiple studies have highlighted the importance of real-time analysis, automation, and adaptive force control to ensure minimal damage during detachment and high viability post-extraction. Huang et al. [17] introduced an automated do-it-yourself (DIY) microfluidic system for stem cell and organoid culture that enables the selection of multiple fluid media and dynamic environmental control in standard multi-well plates. This modular system demonstrates the feasibility of accessible, programmable platforms for long-term cell culture and manipulation. Deckers et al., 2022 [18], proposed a novel detachment assessment feature using lens-free imaging to monitor the enzymatic harvesting process of adherent cells. Their approach bypasses traditional microscopy by employing real-time optical analysis to determine cell readiness for detachment, contributing to non-invasive monitoring techniques.

In the field of on-chip cell lysis and disruption, Doulgkeroglou and Lammertyn [19] presented a mechanical microfluidic platform with an integrated micropump. Their design allows for accurate application of shear forces to break cells and manage liquids without using chemicals—this method can be used in systems that detach cells using microfluidics. Li and Kularatne [20] provided a detailed overview of current detachment methods and their challenges, highlighting the need for controlling force, managing enzyme exposure time, and improving surface coatings. Their work outlined the limitations of current approaches and suggests sensor-based feedback systems as a promising alternative. Challener [21] focused on the bioprocessing industry and discussed harvesting challenges for adherent cell cultures in large-scale systems. The article presented industry-standard practices while also highlighting novel approaches that increase yield and maintain cell functionality during extraction.

Ding and Li [22] designed a 3D-printed modular microfluidic system capable of continuous cell harvesting. Their work emphasized scalability, real-time adaptation, and the integration of customizable modules for industrial or research use, offering insights into cost-effective and replicable systems. Finally, Bose [23] highlighted new developments in cell harvesting, including special coatings that respond to surfaces, flexible materials, and AI-based tracking systems. These technologies aim to reduce mechanical stress and enable precision timing in extraction protocols, aligning closely with the goals of the present study.

Recent research has noted the success of advanced image processing sensors and algorithms in the field of microfluidics and cell analysis [24,25]. In the recent literature, applications of deep learning-based methods have shown promising results in analyzing microscopic images obtained from microfluidic devices [26,27].

The authors in [28] created a system called YOLO-PBESW, which is built on the YOLOv8 design, to accurately recognize the shapes of Indomethacin crystals in tiny droplets. The authors have proposed an algorithm based on an improved YOLOv8 architecture used for the detection of small-sized objects by utilizing high-resolution P2 layers and BiFPN structures. These are intended to facilitate multi-scale information extraction. The authors also added an EMA mechanism to enhance the global attention of the network and used SimSPPF to reduce inference time. According to the experiments, the authors obtained a *mAP* of 87.6% and an excellent detection speed at around 77 FPS. It was observed that automatic detection of small-sized crystals is essential for the pharmaceutical field.

In another study, the authors in [29] proposed a method for automatic segmentation of contacting cells using images obtained from microfluidic chips. The research of this work was focused on concave point detection and matching. The authors initially proposed a UNet++-based deep neural network for identifying cell contours using a dataset combining bright-field and fluorescence imaging. Subsequently, an improved method was developed, focusing on the area of concave point detection using the convex envelope defects of the contours. The study addressed several limitations of existing methods by using a well-targeted strategy that achieved an accuracy of over 90%. The methods addressed include a simple and efficient concave point matching strategy based on a compact measure to ensure accurate segmentation of the contacting cells. The authors in [30] proposed another innovative system based on the YOLOv5 architecture for the identification and long-term monitoring of coli bacteria in microchannels, using microspheres as a positioning landmark. Feature extraction methods from images based on intelligent algorithms that solve the problems of focusing bacteria on multiple layers are presented. The proposed architectures are essential for real-time monitoring, which are ideal for antibiotic susceptibility testing. The authors used microspheres mixed with bacteria in a microfluidic chip to facilitate fast and accurate localization and relocation of cell layers on the z-axis, thus avoiding defocusing problems. The experimental results showed very good performance with an accuracy of over 70% on the cell detection and counting side, highlighting the capability of deep learning architectures for fast and accurate analysis of microscopic phenomena. Collectively, these studies confirm the need for multi-layered control systems that integrate microfluidics, sensors, and smart algorithms. They form a strong foundation for the proposed solution in this paper, which aimed to detect and respond to the pre-detachment moment with minimal perturbation to adherent cells.

## 3. Materials and Methods

This section explains the tools and methods used in the study, focusing on important parts like microfluidics, micromechatronics, sensors, main modules for data analysis and digital image processing, the neural network designs for identifying and segmenting cells, the dataset used for training and testing, performance measures, and the hardware and software that made the experiments possible.

### 3.1. The Microfluidic–Micromechatronic System

**Figure 1:**
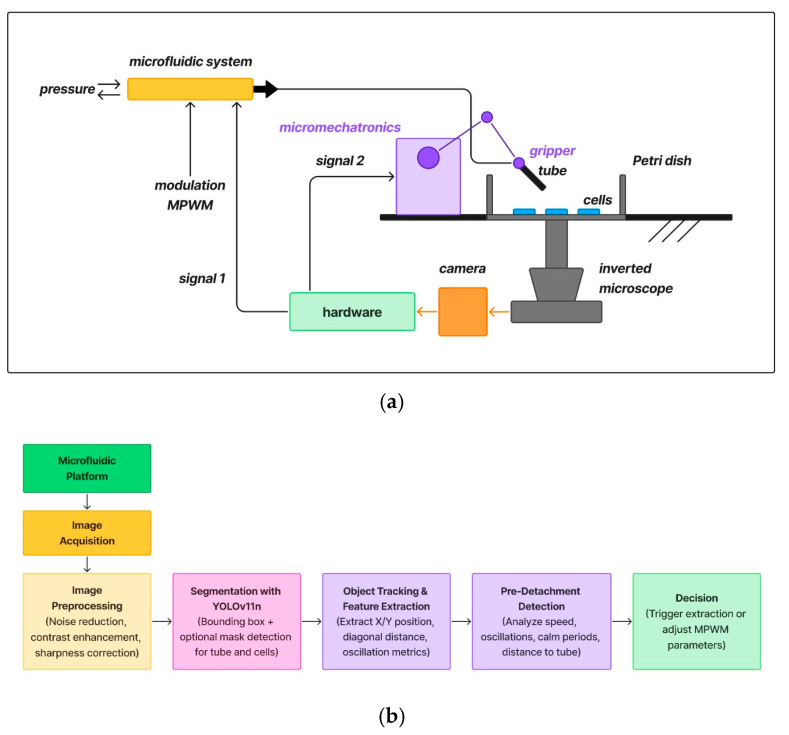
(**a**) The proposed micromechatronic system; (**b**) the data processing workflow diagram.

We specifically designed an advanced microfluidic–micromechatronic system for precise single-cell extraction from cell cultures, intended for detailed biomedical studies. This is shown in Figure 1a. The system mainly includes a microfluidic unit that manages fluid conditions using pressure inputs, which are finely tuned with Modulated Pulse Width Modulation (MPWM). This modulation allows accurate adjustment of suction power and timing, essential for optimizing the extraction of minimal fluid volumes containing individual target cells. Central to the precision of this process is the integration of an inverted microscope equipped with a CCD camera sensor. This camera system provides real-time and static positional information about the cells within the Petri dish, feeding important data back to a dedicated hardware control module. Utilizing the visual feedback from the camera, the hardware unit generates Signal 1, which defines the optimal fluidic parameters required to achieve minimal and precise extraction volumes, thereby enhancing cell recovery efficiency and reducing unnecessary disruption to the cell culture. Complementing the fluidic precision is the micromechatronics module, responsible for controlling a micro-gripper device (tube). Guided by Signal 2, the micro-gripper performs accurate positioning and handling tasks at microscopic scales, enabling targeted single-cell extraction directly from the cell culture in the Petri dish. The cohesive interaction between the microfluidic control, micromechatronic positioning, and visual feedback from the camera system establishes a closed-loop operation, significantly enhancing the reliability and precision of single-cell isolation. It is important to note that the microfluidic working regime was not modified at the level of the filling and pressure factor, but only at the number of extraction–suction pulses.

The MPWM parameters were injector signal frequency 100 Hz, signal duty cycle 30.3%, pressure −370 mbar, and a proportional step number. Images were acquired using a Raspberry Pi Camera Module 3 NoIR (SC0873, Raspberry Pi Foundation, Cambridge, UK) capable of capturing up to 4608 × 2592 pixels resolution with variable frame rates depending on the output mode. For this experiment, frames were recorded at 1920 × 1080 resolution at 30 FPS under bright-field illumination conditions.

Overall, this integrated approach ensures accurate, minimally invasive extraction and separation of individual cells from their culture environment, facilitating precise biomedical analysis and experimentation at a single-cell level. The workflow diagram is shown in Figure 1b, which summarizes the end-to-end data processing pipeline, obtained from image acquisition and pre-processing to detection, evaluation, and post-analysis. This modular pipeline integrates classical image processing with deep learning and real-time control logic, offering a robust and adaptable framework.

### 3.2. Image Processing and Data Analysis Module

In this paper, a methodology for pre-processing and enhancing original RGB microscopic images was described and implemented before being used in the creation of a dataset dedicated to object segmentation. The main goal of this step was to reduce noise, adjust possible sharpness issues, and improve contrast, while maintaining the essential information in the retrieved images.

In the first step, each image was analyzed to determine the degree of sharpness. Beyond the approaches that transform the image into grayscale, we opted for extracting the light channel (L) from the Lab color space. Each RGB image was transformed to Lab, with the Laplacian variance on the L channel chosen as the method evaluating the level of blur and then returning to the initial form. If this variance was below a set threshold (for example, 100), the image was considered blurred. We applied restoration techniques to recover details in this case. An unsharp mask filter was applied to the separate channels of the RGB image, or a deconvolution method using a point response function (PSF) was used when there was some information about the type of blur, whether it was from motion or focus.

In another step, the detection of noise, i.e., extremely bright or extremely dark pixels distributed randomly, was pursued. The method was applied separately for each RGB channel, tracking the weight of the extreme values. A 2D median filter was applied, preserving the color relationships between the channels.

Another important part was the contrast evaluation and adjustment. Avoiding converting the image to grayscale, the Lab space was chosen again for the application of an adaptive CLAHE algorithm on the L channel without affecting the color channels. Such a strategy allowed us to highlight the structural details in the microscopic images and avoid unwanted changes in the chromatic shades. For situations where the contrast was already high, CLAHE was not necessary, thus avoiding possible over-enhancement.

Although careful filtering and adjustment procedures were applied, some images still showed small noisy regions or artifacts. To correct these, color morphological operations (dilation and erosion on each channel) or a simpler approach, which treats color channels separately but synchronizes operations at the corresponding pixel level, were applied. These morphological steps helped to smooth out areas and eliminate isolated points that did not represent relevant biological structures. Finally, each pre-processed image was visually inspected to validate the performance of the implementations and to confirm the existence of key information. The result was a set of RGB images, free of artifacts, with low noise, sharpness, and optimally adjusted contrast. The methodology adopted ensures both data quality and the faithful preservation of relevant information for microscopic analysis. In the scientific approach, this set of digital images was used to generate labels for a segmentation algorithm and to analyze the data and behaviors of the extraction process. As a complementary part of the study, the implementation of a segmentation algorithm using a deep learning architecture was also chosen.

### 3.3. Dataset Used

**Figure 2:**
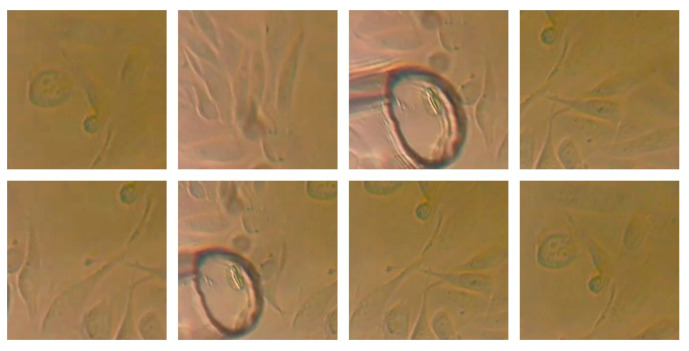
Example images from the collected dataset.

A fundamental part of a deep learning system capable of extracting relevant features and information from digital images is a robust dataset [31,32]. Organizing a digital image dataset is an important step in the context of training and evaluating deep learning models for identifying, localizing, and segmenting cells in digital images. A central element of the present study was the organization of such a dataset, carefully collected and pre-processed to ensure adequate representativeness of the diversity in clinical applications and to dictate the success of a deep learning model learning from these data. The dataset contains a total of 950 digital images, which capture the natural variability of shapes, sizes, and signal intensities. The image set was divided into three subsets—train, validation, and test—according to standard practices in deep learning, ensuring variability and accuracy of the evaluation. The testing part was used to evaluate the performance of the model on new images. The division of the dataset was performed according to the following ratio: 70% training (summing 665 images), 20% validation (summing 190 images), and 10% testing (summing 95 images). Figure 2 contains example images from the collected dataset.

To maximize the performance of the deep learning model and prevent overfitting, the images collected for the dataset included data augmentation techniques to help increase the generalization of the deep learning model. These help the model become more robust to real-world variations in the images. The augmentation techniques involved different changes made to the digital images, including flipping them (both horizontally and vertically), rotating them 90° (upside down), rotating them slightly between −15° and +15°, adjusting saturation between −25% and +25%, changing brightness between −15% and +15%, modifying exposure between −10% and +10%, and adding noise to up to 0.25% of the pixels. Good image quality, avoidance of bias, relevance of data to the chosen tasks, and attachment of quality labels by professionals in the field were important steps in creating the dataset for the present study. All these implementations ultimately contributed to increasing the diversity of the dataset, reflecting the varied conditions of image capture.

### 3.4. Neural Networks Used

As part of this study, a model from the YOLO (You Only Look Once) family was used to identify and segment cells in digital images. The architecture chosen for implementation in the present study was Ultralytics YOLOv11 [33]. YOLOv11 represents a recent evolution of the YOLO model series, bringing significant improvements in real-time object detection and segmentation compared to previous models. YOLOv11 is distinguished by a highly optimized architecture that combines high accuracy with computational efficiency. This makes the model suitable for a wide range of computer vision applications. Specifically, YOLOv11 is designed to be versatile, supporting multiple computer vision tasks, such as object detection, instance segmentation, image classification, and object detection in various orientations. This adaptability makes it ideal for various domains, including medical and biomedical applications.

Among the key innovations of the new YOLOv11, the introduction of the C3k2 (Cross Stage Partial) block, which is designed to improve the extraction of essential features from digital images, has been noted to allow more precise detection and high accuracy. On the other hand, the model also uses an SPPF (Spatial Pyramid Pooling–Fast) component to efficiently manage information in different orders of magnitude, increasing the ability to detect objects of different sizes. The integration of a C2PSA (Convolutional Block with Parallel Spatial Attention) module helps direct the model to a series of relevant regions of the image, thereby improving overall performance. This efficiency allows the model to be deployed on diverse devices, such as edge, cloud, and systems with significant hardware support, ensuring maximum flexibility in various operational environments.

Following the details presented above, YOLOv11 stands out for introducing new standards in real-time object detection and segmentation, integrating advanced feature extraction techniques and optimizing computational efficiency, being useful for research in the present study. The implementation details followed the official documentation of the YOLOv11 model, as well as the details attached to the official GitHub page [33].

YOLOv11 was chosen for the balance between detection accuracy and real-time efficiency, essential for our cell imaging workflow. Although alternative architectures, such as Mask R-CNN, could offer detailed instance masks, the bounding box and segmentation outputs from YOLOv11 were sufficient for our investigation, facilitating faster inference and simplified implementation in real-time or embedded contexts. Where higher precision masks are needed, lightweight segmentation refinements can be added on top of YOLO outputs. On the other hand, YOLOv11 has a lighter architecture and is easier to deploy on resource-constrained systems (e.g., embedded GPUs like NVIDIA Jetson), while alternative architectures require significantly more memory and computation, which may not be feasible for high-throughput cell analysis.

### 3.5. Hardware and Software Used

The implementation and evaluation of the deep learning model were facilitated by a robust custom system, based on an Ubuntu v22 operating system. From a hardware perspective, the experiments were carried out on a state-of-the-art platform, essential for training complex neural networks. The setup was described by an Intel Core i9 14900HX with 36M Cache, 32 GB DDR5 RAM, 1 TB SSD, and a high-performance GeForce RTX 4090 16 GB GPU with CUDA support. From a software perspective, the development environment was based on established open-source frameworks, such as PyTorch v2.7. The chosen programming language was Python v3.11. These provided the necessary flexibility for implementing and adjusting the models. In addition, specialized libraries for image processing and data manipulation were also used. This ensures optimal data flow management and reproducibility of the experiments. The hardware and software configurations allowed for parallel execution of processing operations, reducing computing times and increasing training efficiency.

### 3.6. Performance Metrics

The evaluation of the models used for cell segmentation was performed using a set of standardized metrics in digital image analysis, which faithfully reflect both detection performance and segmentation accuracy [34]. Table 1 presents the relevant performance metrics, including the corresponding mathematical formulas.

*mAP* (Mean Average Precision) is a standard in the evaluation of detection and segmentation models. This indicator provides an overall measurement of the model performance under different overlapping conditions (*mAP@0.5* or *mAP@[IoU = 0.5:0.95]*), calculating the average precision for different thresholds of the Intersection over Union (*IoU*) coefficient. *IoU* describes the ratio of the overlapping area to the total area of two regions (model segmentation vs. ground-truth). For a segmentation task, a higher *IoU* indicates a more accurate overlapping area. Typically, *mAP@0.5 (IoU ≥ 0.5)* is a more permissive option, while *mAP@[0.5:0.95]* is stricter and better reflects overall performance. In Table 1, for *mAP* formula, ${AP}_{i}$ is the average precision for each class and *N* is the total number of classes evaluated. For *IoU*, *A* is the surface segmented by the model, *B* is the reference surface (ground-truth), A∩B is the intersection of the two regions, and A∪B is the union of the two regions. The *mAP@[0.5:0.95]* metric is unitless and reported as a value between 0 and 1, following the COCO evaluation protocol.

*Precision*, *Recall*, and *F*1 score are used to determine the ability of the model to detect cells without introducing false positives or significant omissions. Precision describes the percentage of correct detections out of the total number of predictions made, while Recall describes the percentage of correctly identified objects out of the total number of existing objects. The *F*1 Score represents the harmonic mean between Precision and Recall, providing a balance between the two and a good metric to investigate the performance of a chosen model. These scores can be extracted from the definition of the so-called confusion matrix, ensuring a complete analysis of the accuracy and precision of the algorithms, where indicators such as *TP* = True Positives, *FP* = False Positives, *FN* = False Negatives, and *TN* = True Negative are used. Calculating these metrics gives a thorough assessment of the model, making it possible to compare its performance with other methods and studies in the area, while also offering a strong basis for enhancing the algorithms [35,36,37].

## 4. Experimental Results

This section presents the results obtained based on the digital image processing modules and attached sensors. The results of training the YOLOv11 model for cell segmentation from the customized dataset are also presented. For the segmentation part, this study tested how well the model works in different situations, highlighting how effectively the algorithm can find and accurately separate cells that come in different shapes and sizes. In the image analysis area, the experiments and modules aimed to understand how cells behave in microfluidics culture and how well the CCD sensor and software work together to produce useful data for the application.

The present study follows and analyzes the behavior of a cell extraction process from various cultures. Using a collection of images that were prepared beforehand, we tracked how the cell extraction process changed over time by looking at a series of microscopic images that show how the cell population reacted during different attempts to separate the targeted cells. We used several example frames in this study, numbered from the initial time *T_start* (first image) to the final time *T_end* (last image). Each frame illustrates a part of the extraction process, and the effects of the extraction attempts are monitored to observe the impact on the other cells in the field. A chosen example was represented by 395 images, where *T_start* = 0 and *T_end* = 394. To manage and analyze this process, a data analysis module was developed using the Python language, which is very popular for these cases. The module was designed to automatically analyze all the images prepared for the present study, being able to extract geometric and positional information about the cells of interest. The module tracks changes over time and the effect of extraction attempts on each cell, making their position and shaping key parameters. Using the implemented module, the position of each cell, its framing area, and the coordinates of the center (X center and Y center) are determined. Excel files were chosen as the recording medium for management, as they facilitate both subsequent data analysis and their attachment to graphics or data analysis applications. For each image, the generated table contains one row for each detected cell, including the information necessary for the position of each cell in columns, such as the image index, the detected object name, the X and Y coordinates, or the X and Y centers.

All spatial measurements (e.g., position, displacement, diagonal length, and inter-object distance) are expressed in pixels (px), as derived from the image coordinates of the object bounding boxes and centroids. Time is expressed in frame units, where one frame corresponds to a single image in the video sequence.

As the process takes place and extraction is attempted, some cells change their position or shape, either in response to direct intervention or because of natural adjustments in the culture medium. Therefore, the implemented Python module regularly captures these changes, ensuring temporal accuracy. This analysis method allows the observation of the spatial relationship between the extracted cell and other neighboring cells. For example, intercellular distances may increase or decrease in certain regions, and certain cells may reorient or disperse depending on local conditions. At the same time, the collected parameters serve as a basis for interpreting the phenomena of cell adhesion, migration, or redistribution. This method helps create strong tools to monitor how a group of cells changes over time and makes it easier to find links between the extraction event and how the other cells respond. On the other hand, the history of each cell can be followed with the potential to correlate data with certain biological, adhesion, mechanical, or biochemical aspects.

**Figure 3:**
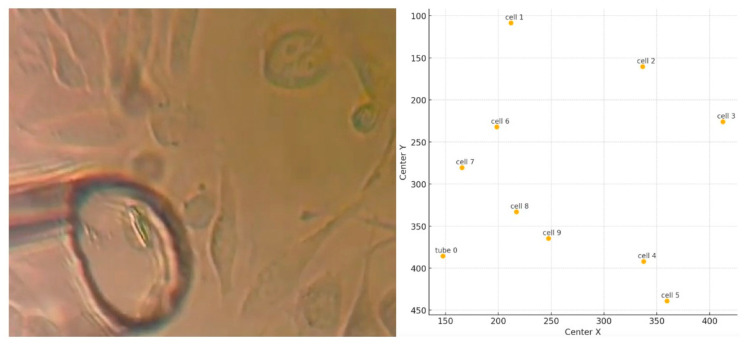
Illustration of the process and the position of objects of interest.

The current instance features an extraction tube (designated as object 0) attempting to remove a cell from a culture. Numerous cells exist, with the cell designated as item 9 for extraction. Objects are represented by the numerals 0 through 9 (Figure 3). The method is iterative, requiring several tries and modifications to the tube’s location to attain a successful extraction. Cell extraction takes place at frame 352 among the 395 sample frames.

Considering these aspects, image processing and data analysis have a notably significant role. This framework exhibits several features: object identification and localization, trajectory computation based on position and size, velocity assessment and oscillation detection to identify anomalous behaviors, and evaluation of tranquil intervals, signifying periods of diminished or absent movement. Various graphs and statistical approaches were employed to detect abnormalities in the investigation of object movement and suspicious behaviors. These approaches integrate location analysis, velocity detection, and tranquil interval analysis, thus offering a comprehensive comprehension of the observed occurrence. The extraction procedure and the movement of items include several observable steps. Each phase is characterized by distinct behaviors of both the tube and the cells.

The first stage is marked by the initial positioning of the tube, strategically, in the proximity of the cell to be extracted. The cell is in a period of calm, with minimal, negligible movement. The trajectory of the tube is controlled and precise in trying to analyze an optimal position for extraction. In Figure 3, the tube is positioned in the proximity of cells 8 and 9. Cell 9 is the one that is desired to be extracted.

During the described process, there will be several extraction attempts. During this time, the tube adjusts its position and tries to extract the targeted cell. As soon as the movements become more intense, the cell starts to show unpredictable reactions, affecting the cell field and, consequently, the other nearby cells.

**Figure 4:**
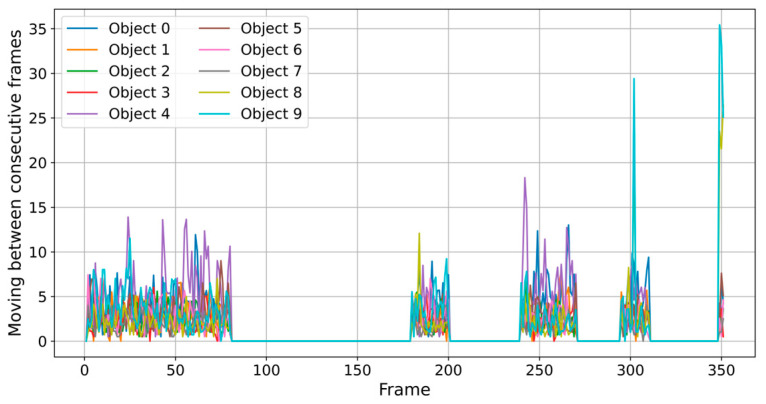
Model for oscillation and calm period (units: oscillation in pixels (px); time in frames).

**Figure 5:**
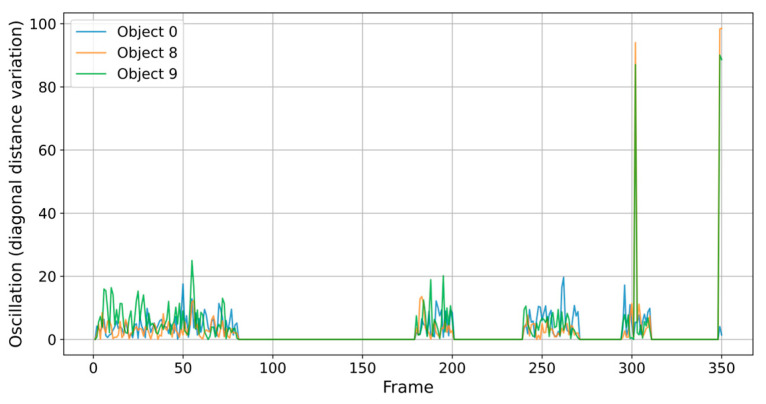
Model for oscillation and calm period for objects with the most visible evolution.

Figure 4 and Figure 5 show oscillation patterns and the quiet periods in this process. Oscillations represent frame-to-frame differences in diagonal size or position and are measured in pixels. Diagonal Distance reflects the Euclidean length of the object bounding box diagonally and is measured in pixels (px). It is interesting to note the quiet periods, where the cells hardly change their position, occurred immediately after the extraction attempts that result in the oscillation periods. According to these data from the graphs, object 9 is suspicious in its evolution, being in fact the cell that is to be extracted. The major changes at frames 300 and 350 suggest moments of abrupt transition.

**Figure 6:**
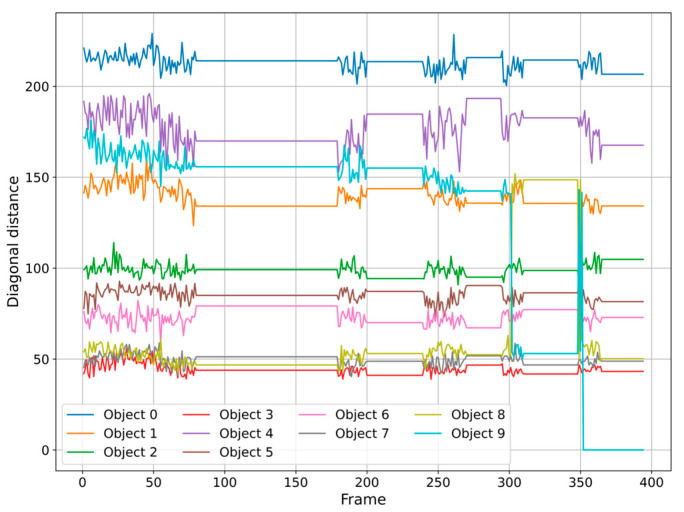
Evolution of diagonal distance for each object (units: diagonal length in pixels (px); time in frames).

**Figure 7:**
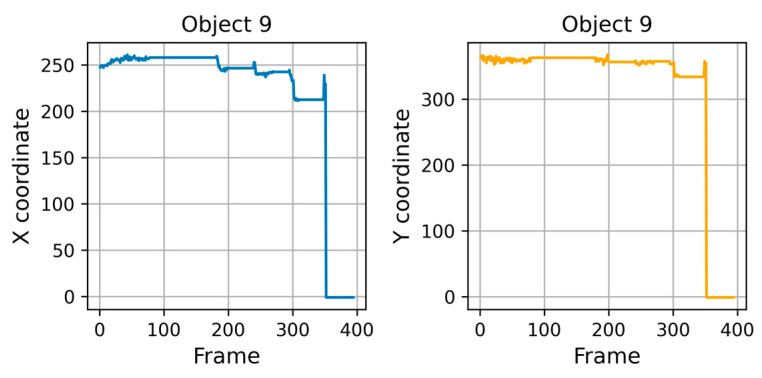
Viewing the evolution of X and Y coordinates for object 9 (units: X and Y positions in pixels (px); time in frames).

In Figure 6, we can see how the diagonal distance changes for each object, showing times of silence and movement while trying to obtain the reference cell. These attempts affect the cellular field, causing the cells to change their shapes and positions. The extraction attempts clearly influence the cellular field, causing the cells to modify their shapes and positions. We observe a sudden drop-off effect when we extract cell 9 at frame 352 using the tube. The distance evolution exceeds a critical threshold, and the cells reorient slightly after this successful extraction. Analyzing the X and Y coordinates of the extracted cell also reveals this evolution. Figure 7 illustrates this evolution by isolating the suspect object and observing the gradual decrease that results from multiple extraction attempts.

**Figure 8:**
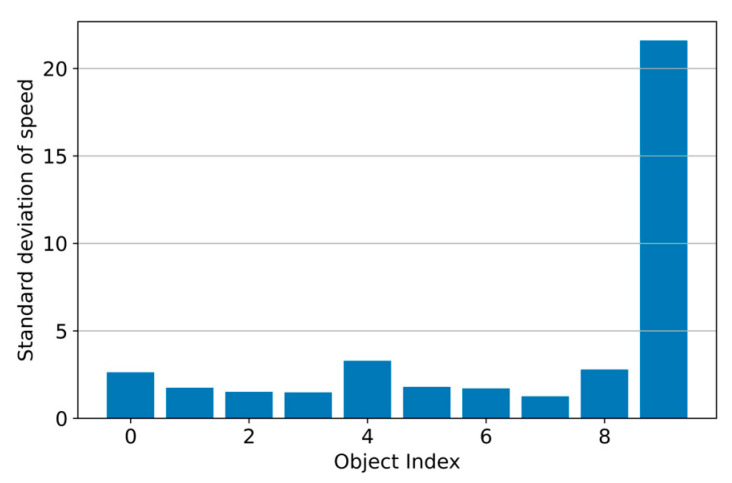
Comparing velocity variability between objects.

We noted that the extraction attempts propagate these forces in a cell field. The behavior is confirmed by the significant increase in the cell velocity at different distances from the tube and by the appearance of several oscillations (Figure 8). We used the Euclidean norm to calculate the positional variations between consecutive frames to analyze the object’s velocity. Object 9 is suspicious due to its sudden oscillations, erratic movements, and large variation in velocity compared to other objects (pixels per frame). An unusual movement or change in position, not typical of other objects, demonstrates this.

**Figure 9:**
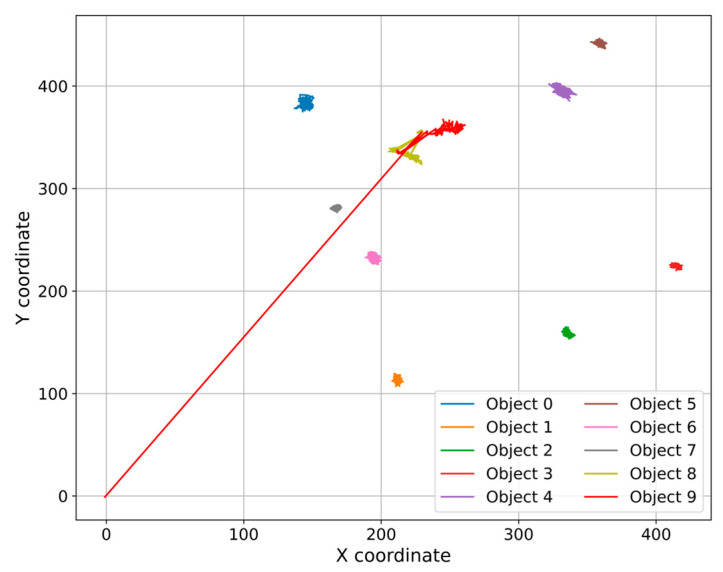
2D plane trajectory for objects 0–9 (units: position in pixels (px) on both X and Y axes).

**Figure 10:**
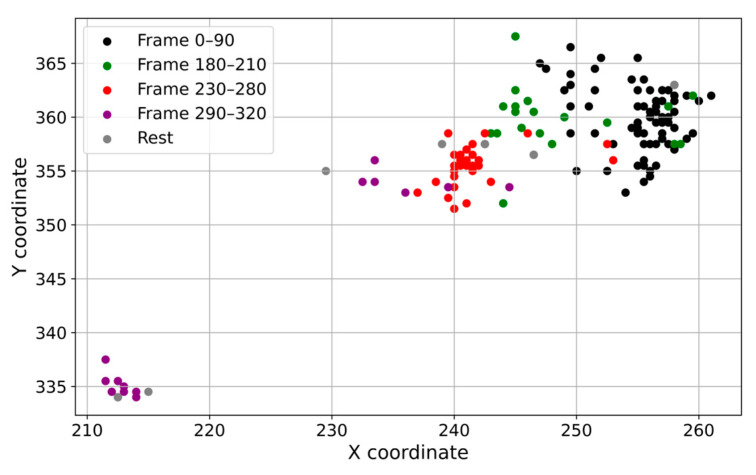
2D plane trajectory for Object 9 (units: position in pixels (px) on both X and Y axes).

Following the tube and establishing a firm position to extract the cell, the cell exhibits rapid oscillations and significant dispersion of its positions. The forces propagate within nearby cells. Once the final stage of the process, implicit extraction, occurs, the cells in the field exhibit lower fluctuations, indicating either a successful final attempt or stabilization. This also occurs when previous attempts fail, with the stages of calm being described as plateaus of calm. The trajectories and positions in the 2D plane for the cells in culture and for the extracted cell 9 are shown in Figure 9 and Figure 10. The 2D trajectory plot was illustrated in this case to analyze the movement of objects in a two-dimensional space. This method clearly highlights accumulation areas, frequent movement directions, and abnormal behaviors.

**Figure 11:**
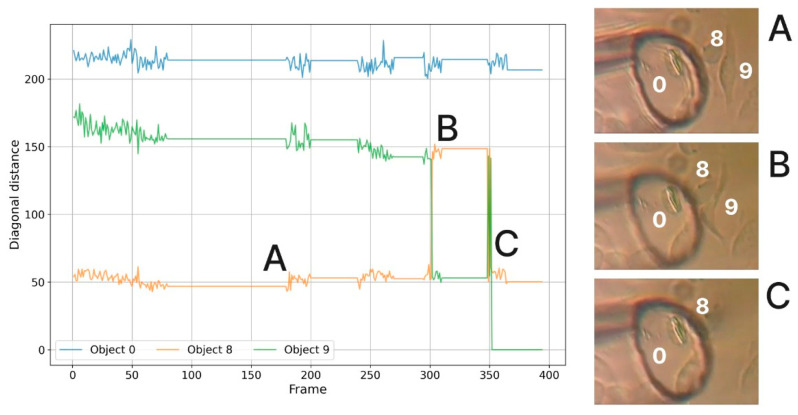
Exemplifying extraction moments (units: diagonal length in pixels (px); time in frames).

Figure 11 presents an analysis of how the data evolve throughout the process and how the images on the right reflect the corresponding states in each region of the graph.

The plot in Figure 11 shows the diagonal length evolution of three tracked objects (Object 0, 8, 9) during extraction. Point A marks the initial pre-detachment moment, with minimal diagonal fluctuation across all tracked objects. Point B indicates a sudden increase in diagonal length for Objects 8 and 9, suggesting deformation due to suction. Point C shows a sharp drop for Object 9, corresponding to its successful detachment and disappearance from the frame. The corresponding microscopy frames (right) visually confirm the cell configurations at these key time points.

Looking at the previous figure, the algorithm can identify the pre-detachment moment based on several criteria. By viewing the graph between frames 300 and 350, we can deduce this step given the sudden evolution of the diagonal distance. With the help of the algorithm, the pre-detachment moment is identified at frame 348. The moment at frame 348 is close to frame 352 (the actual moment of extraction), suggesting that the tube maintained a stable position for a short period before the final force was applied. The distance between the tube and the cell is optimal for extraction (estimated to be less than 90 px).

Regarding the deep learning area, the approach was based on the use of the transfer learning technique, through which the YOLOv11 model was initialized with pre-trained weights on a custom dataset presented previously. A fine-tuning stage completed the technique, adapting it to the characteristics of the experimental data. This approach allowed for a reduction in the training time and improved the model’s ability to identify complex details of the cells.

The YOLOv11 model was trained on microscopic images utilizing a high-quality dataset, where each cell was meticulously labeled, particularly for segmentation, with a mask or polygon outline. These labels were acquired through manual annotation by experts in the field to guarantee the utmost level of precision. Two classes were indicative of the YOLO segmentation model: tube and cell. Conversely, several parameters were selected to conduct the experiments utilizing the YOLOv11 architecture: Training was to be conducted for 100 epochs with a batch size of 32, utilizing 8 workers. The learning rate was set at 0.001, employing the SGD optimizer with a momentum of 0.9. The YOLOv11n model was selected for implementation, aiming to deploy it on hardware with constrained resources. The training of YOLOv11 followed an iterative methodology, with each epoch consisting of a forward pass on the training set. During this phase, the model generated predictions for bounding box structures and masks. This was followed by an error calculation, which involved comparing the predictions with the actual labels, and a backward pass to modify the weights aimed at minimizing the error.

**Figure 12:**
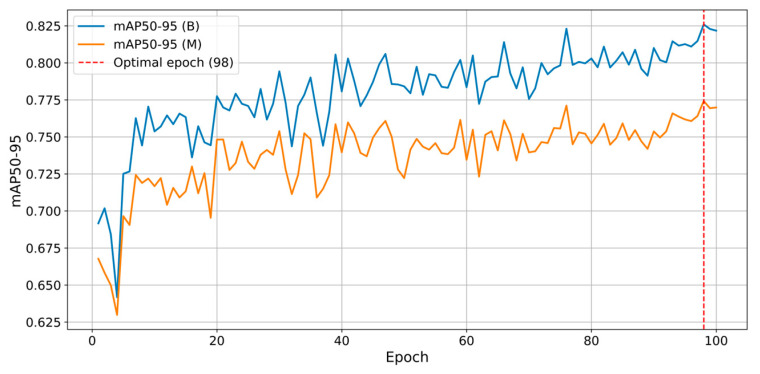
Evolution of *mAP50-95* with highlighting of the optimal epoch.

**Figure 13:**
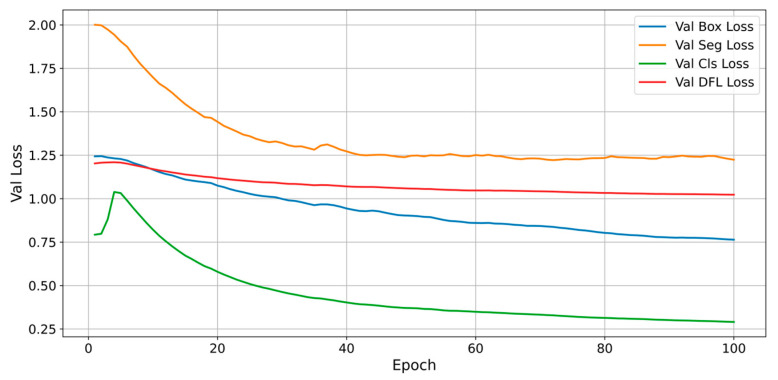
Evolution of validation losses.

The results obtained from the adopted YOLO architecture underscore the effectiveness of the methods proposed in this study. The results achieved with this deep learning architecture were highly satisfactory. Figure 12 illustrates the progression of *mAP* metrics for the YOLOv11 model that has been trained. Figure 13 illustrates the progression of validation losses. The current study revealed impressive outcomes of the YOLOv11 model regarding segmentation and cell identification tasks. The model demonstrates strong performance in object detection, excelling at both the bounding box and mask levels. The data gathered throughout the training and validation phases demonstrate a consistent convergence and a notable enhancement of the loss function, alongside elevated *mAP* values. The results obtained indicate the model’s capability for application in scenarios demanding rapid and precise cell identification and segmentation.

The evolution of the losses and performance metrics reflects both the model’s ability to learn efficiently and its stability and generalization aspects. The total loss (weighted sum of the losses for localization, classification, segmentation, and anchor distribution) decreases steadily over the epochs, indicating a progressive convergence of the model. However, in the first epochs, the classification loss shows a pronounced peak, a sign of a temporary instability. The segmentation loss maintains a relatively high level, with slight fluctuations. This aspect may indicate difficulties in identifying fine details of the cells or a problem in the total loss function. The development of advanced modules for the YOLOv11 model could improve this behavior. At the same time, this decreasing trend of the loss functions confirms that the model not only memorizes but also generalizes quite well.

**Figure 14:**
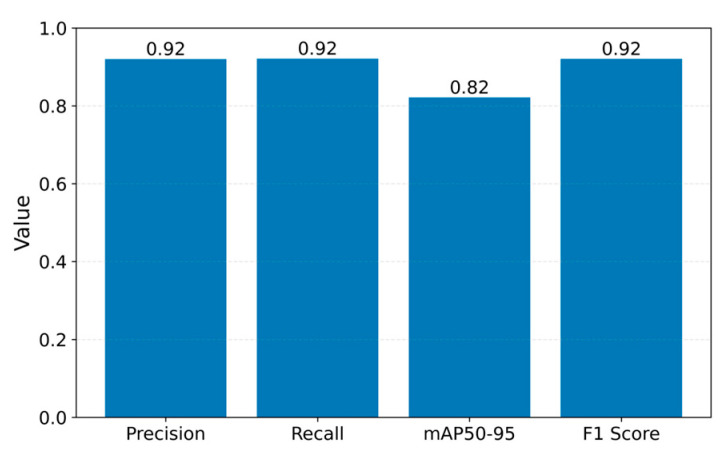
YOLOv11n metrics.

Another key indicator is the balance between precision and recall, both at the bounding box and segmentation levels. This is shown in Figure 14. Towards the end, the model reached values above 0.92 for both precision and recall. This suggests that the YOLO model manages to correctly identify most cells while avoiding many false positive detections. The performances are supported by an *mAP50* of approximately 0.915 and an mAP50-95 of approximately 0.77, values that indicate that, at higher *IoU* thresholds (e.g., 0.7 or 0.8), the model maintains a high level of accuracy for segmentation. The high values of *mAP* and *F*1 *Score* confirm the model’s ability to generalize effectively.

**Figure 15:**
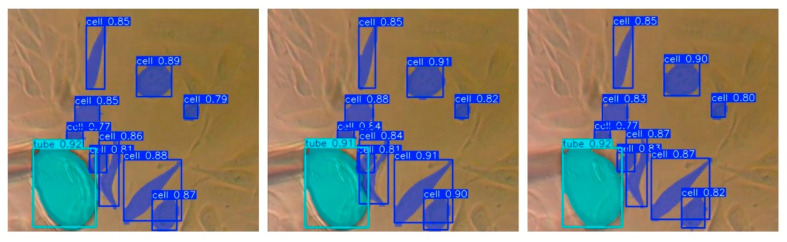
Examples of segmented cell predictions using YOLOv11n.

In addition to the numerical evaluation, visual results are also presented to illustrate the clarity and accuracy of the segmentations. Figure 15 shows examples of segmented cells, close to the object represented by the extraction tube. They highlight both the strengths of the model and the challenges encountered, such as incomplete segmentation with partially overlapping cells or incorrect identification of artifacts as cells. Columns 2 and 3 of Figure 15 capture the moment of extraction targeting the cell represented by the previously described object 9, from which it is observed that the extraction process completely modifies its shape and position.

## 5. Discussion

In this paper, the analysis of the evolution of the diagonal distance, the oscillation patterns, and the displacement of the objects provides a clear picture of the complexity of the cell extraction process. The precise detection and interpretation of these data allows the smart sensor to identify the optimal moment for extraction, control the applied force, and react adaptively to the unpredictable behavior of the cell.

The integration of CCD, image processing, and deep learning algorithms provides advanced and adaptive control, maximizing the efficiency of the process and protecting the cell from damage. This represents an innovative solution for optimizing cell manipulation procedures in fields such as regenerative medicine, biotechnology, and biomedical research.

For the present case, the system was described to monitor in real time the position, movements, and reactions of the objects involved in the extraction. The extraction tube (object 0) and the target cell to be extracted (object 9) were mainly targeted. Detailed analyses were also made in relation to the rest of the objects to study the impact of the extraction zones. Data analysis provided a complex perspective on the behavior of these cells, and highlighted the role of diagonal distance, oscillations, calm periods, and pixel displacement in determining the optimal moment for applying the extraction force.

The CCD sensor-based system plays an important role in ensuring the accuracy of visual data. The ability to capture images with fine details and a good signal-to-noise ratio has allowed for detailed analysis of objects in digital images, their edges, and precise monitoring of their position. Such a module is essential because variations in position and size can affect precise monitoring and the extraction area. Moreover, with the integration of image processing algorithms and deep learning architectures, the CCD-based system allows for clear observation of the process, precise tracking of objects, and detection of optimal points for extraction.

For the described experiments, the evolution of the diagonal distance of the objects provided valuable information regarding dimensional changes and position during the extraction process. The diagonal distance reflects the effective size of an object in the visual field and is sensitive to any change in position, rotation, or deformation. In the case of the tube (object 0), the diagonal distance had a relatively stable evolution, with minor variations corresponding to fine adjustments of the position during the extraction attempts. This behavior indicated a precise control of the tube and a targeted approach in the attempts to intercept and extract the cell. On the other hand, object 9 (the extracted cell) showed pronounced fluctuations in the diagonal distance. It showed numerous sudden increases and decreases and a pronounced oscillation throughout the process. At the same time, this atypical behavior is specific to cells that, following the application of a mechanical force, change their shape or adjust their position to avoid manipulation or to preserve their natural balance. Identifying these fluctuations was essential because the sensor is designed to detect defensive patterns and automatically reduce the force applied at such times.

Identifying the periods of calm and oscillation was essential for optimizing the extraction process. Oscillations described moments of instability and were defined by the sudden and frequent movements resulting from the extraction attempts. In the case of the tube, the oscillations had a more controlled and less intense character, being defined by the attempt to position the tube for cellular extraction. In the case of cells in culture, especially in the case of the cell that was intended to be extracted, the oscillations occurred in a more pronounced, often chaotic manner. These can be interpreted as defensive reactions of the cells, indicating moments when the extraction force exceeded acceptable thresholds.

The periods of calm, represented by the stillness levels in the graphs attached previously, represented moments in which the objects had minimal, negligible, or non-existent movement. This behavior indicated periods of stability. For cells, these moments indicated periods without defensive reactions and a well-determined equilibrium, stabilizing in the visual field. For the object represented by the tube, these moments are defined for preparing the extraction process and for applying force. Identifying such moments allows the sensor to initiate extraction in safe conditions without mechanically damaging the cells or elements of the culture.

These data can be analyzed to understand the dynamics of movement, deformations, movement patterns, and variations in the amplitude of their movement. There may be a wider spectrum in vertical or horizontal movements. Several observations can take part in these described phenomena. Cells respond to external forces in a balanced way to maintain the stability of the tissue they are part of. Marginal displacement, dilation, and contraction are important variables in this process, analyzing the maximum expansion and contraction on the X and Y axes. Visible connections can be represented by the fact that cells that move more on the X axis tend to move less on the Y axis, so there is a preference for a certain direction. At the same time, the sensor can indicate a dimensional balance for each cell based on dilation and contraction on X or Y. When a cell moves a lot on one axis, it dilates as much on the same axis, with the cell adjusting its size on this axis to return to equilibrium or to maintain its shape. A balance between dilation and contraction may suggest cell modification through a coordinated process or may describe the mechanical stability of the cell, important behaviors captured by the sensor for decision-making.

In this study, successful detachment was defined based on a displacement threshold calculated using frame-by-frame tracking of the cell centroid. Specifically, a cell was considered detached when it exhibited a sudden displacement of more than 85–90 pixels between two consecutive frames, combined with an absence of reappearance or reattachment in subsequent frames. This threshold was established empirically based on repeated observations of detachment events, as shown in Figure 7, Figure 8, Figure 9, Figure 10 and Figure 11. Although this method reliably identifies detachment in our context, it remains a kinematic criterion. In scenarios requiring higher confidence (e.g., for cell viability assays or precise biomechanical studies), integration with additional confirmatory tools such as fluorescent adhesion markers, tensile force microscopy, or electrical impedance spectroscopy could be beneficial. These tools would provide biochemical or mechanical validation of complete detachment, particularly in borderline cases where cells may appear visually detached but remain partially adherent. To minimize the volume of liquid required for extraction, the number of MPWM signal steps was strategically adjusted while maintaining constant pressure and duty cycle, thereby ensuring efficient detachment of the adherent cell during the final oscillation. The primary operational parameter utilized in this preliminary study was the number of steps applied to the MPWM signal, serving as a practical lever through which the operator could modulate the final extraction volume. As cell detachment neared completion, the step count was intuitively reduced by the human operator. Although this study relied on human intuition, this parameter is expected to become the key factor in the future automation of adherent cell extraction.

It was observed that the determination of the pre-extraction moment using the image analysis module is based on the analysis of several key criteria: the exact trajectory in time, the speed variation, the movement and calm intervals, the comparative analysis with other objects, and the trajectory analysis in the 2D plane. By superimposing these data on the speed, the diagonal distance, and the distance from the object represented by the tube, the algorithm manages to detect the pre-detachment moments, alerting the operator for subsequent safe and efficient extractions.

Following detailed analyses of the motion data, the sensor implementation describes a necessary step for various reasons—it detects the optimal moment for extraction in a controlled and intelligent way. At the same time, extraction forces can be managed based on the behavior and reaction of the cell, and operators or the control system will be warned about unsuccessful or dangerous attempts.

The deep learning area complements these techniques by using a detection and segmentation model of the YOLO architecture. The obtained performances demonstrate that the YOLOv11 model has a solid learning and generalization capacity, marking the possibility of its implementation in practical applications.

We acknowledge the potential benefit of integrating complementary tools to confirm cell detachment, particularly in borderline cases. Regarding measurement accuracy and confirmation tools, as this is a preliminary investigation, the primary focus was on evaluating the feasibility of image-based detection of pre-detachment events using a YOLOv11n segmentation model and data analysis module. All measurements were based on high-resolution imagery captured using a calibrated camera. While the current method relies solely on frame-to-frame displacement and visual analysis to determine detachment, we acknowledge that this kinematic-only criterion may not capture more nuanced cell–surface interactions (e.g., tethering, rolling, or partial adhesion loss). Future implementations of this system could benefit from the integration of additional sensing layers, such as fluorescence markers targeting adhesion proteins, electrical impedance spectroscopy to detect detachment through resistance changes, and optical coherence microscopy or traction force microscopy for real-time biomechanical validation. Such tools would provide independent and possibly more sensitive confirmation of cell detachment, helping to distinguish between complete and partial release.

The performance metrics presented for the segmentation task (e.g., true positive, false negative, *mAP*, *F*1 score) were computed directly by the YOLOv11n training and validation pipeline. The model compared its predictions against the ground-truth annotations Although this provides a reliable framework for evaluating detection and segmentation, we acknowledge that additional tools (e.g., impedance-based sensing or fluorescent adhesion markers) could further enhance the confirmation of physical detachment events.

Unlike conventional segmentation tasks where cells are imaged in static environments or post-detachment states, our study targeted the specific and transient pre-detachment moment of adherent cells within an MPWM microfluidic extraction system. This represents a unique challenge due to the subtle morphological cues, variable forces, and dynamic fluid–structure interactions involved. As such, we found limited prior work addressing this exact problem, making direct performance comparisons difficult. To the best of our knowledge, there are few to no existing studies that focus specifically on the detection or segmentation of adherent cells during the pre-detachment moment within an MPWM (Microfluidic Pulse Width Modulation) extraction system. This limits the availability of directly comparable benchmarks in terms of detection accuracy or segmentation performance in such a specific biological and technical context.

Our current model was trained and validated on a specific cell dataset, and while initial tests suggested good adaptability, we acknowledge that generalization to morpho-logically distinct or rare cell types may require fine-tuning or retraining with additional annotated data. This requirement is particularly relevant for applications involving diverse cell sizes or imaging modalities. The proposed system for segmenting and analyzing data is flexible and can be adapted for diverse cell types. Furthermore, applying the method in diverse contexts such as different imaging modalities or experimental setups (e.g., 3D cultures, microfluidic systems) could introduce variability that impacts performance. Nevertheless, the architecture’s modular design and compatibility with transfer learning facilitate adaptation to such variations, making it a promising candidate for broader biomedical applications. Although YOLOv11 offers improved speed over earlier models, latency remains a consideration in strict real-time scenarios, especially when high-resolution input frames or multi-camera setups are involved. In scenarios such as high-frame-rate video capture, embedded systems (e.g., Jetson Nano, Raspberry Pi), or multi-stream analysis, latency introduced by pre-processing, inference, and post-processing can accumulate. Further optimization (e.g., pruning, quantization, or deployment on high-performance GPUs) may be necessary for seamless integration into real-time cell tracking systems. Future work could enable deployment in time-sensitive applications like live cell tracking or automated microscopy.

Although the dataset includes 950 annotated images across diverse time points and cellular behaviors, we acknowledge that this quantity may be insufficient to fully capture the heterogeneity of cell types, motion patterns, and detachment conditions encountered in broader applications. This limitation is partially addressed through extensive data augmentation (e.g., rotations, flips, brightness and contrast shifts), which improves model generalization during training. Nevertheless, expanding the dataset with additional cell types, imaging modalities, and experimental conditions could be a next step to further improve model robustness and broaden applicability.

## 6. Conclusions

The main objective of this work was to develop and validate an innovative method for detecting the moment of pre-detachment of adherent cells, using short movies resulting from the exploitation of a semi-automated micromechatronic–microfluidic integrated system. The focus was on real-time monitoring of cell behavior within an MPWM extraction circuit with the aim of reducing mechanical stress on cells and maximizing their viability in biomedical applications.

The proposed system integrates several essential components: a micromechatronic actuator (injector component), a microfluidic aspiration channel with pressure control, MPWM filling factor with a number of suction pulses, a CCD-based video capture module, as well as a software component for real-time image analysis. Detection of the pre-detachment moment is based on the analysis of cell positional oscillations and changes in adhesion dynamics. Image processing is assisted by artificial intelligence algorithms (convolutional neural networks) that identify the geometric center of the cell and track its variation over time. By dynamically adjusting the previously listed microfluidic parameters oriented towards aspiration, based on this feedback, the system optimizes extraction without compromising cellular integrity. The proposed methodology paves the way for the implementation of an automated system with feedback loop on image processing and interconnection with microfluidic parameters.

Experimental results demonstrated that the proposed method can successfully detect the pre-detachment phase with high sensitivity. According to the analyzed materials, cells on the verge of detachment exhibited characteristic oscillatory patterns, quantified by the coordinates of the center of mass and their rate of change. The segmentation and classification algorithms achieved an accuracy of over 90% in recognizing cells and tracking their behavior.

By integrating adaptive fluidic control, digital imaging, and artificial intelligence, this system will contribute to the development of a robust platform for the extraction of adherent cells under minimally invasive conditions. The implications are particularly important in the fields of regenerative medicine, single-cell research, and biotechnology. In this regard, the system could be extended for use with sensitive cell cultures (e.g., stem cells) and adapted for automated operation in a laboratory or production line.

For further development of the work on the deep learning and image processing side, the aim is to expand the dataset by including additional images to cover a wider variety of scenarios and experimental conditions. In addition, new or combined neural architectures can be tested, which include advanced mechanisms to increase the robustness and generalization of the model. Finally, the methodology can explore methods of image augmentation and synthetic generation to improve the performance of cell detection and segmentation.

## Figures and Tables

**Table 1:** Performance indicators.

Indicator	Formula
Intersection over Union (*IoU*)	$IoU=\frac{\left|A\;⋂\;B\right|}{\left|A\;\cup \;B\right|}$
Mean Average Precision (*mAP*)	$mAP=\frac{1}{N}\sum_{i=1}^{N}{AP}_{i}$
Precision (*P*)	$P=\frac{TP}{TP+FP}$
Recall (*R*)	$R=\frac{TP}{TP+FN}$
*F*1 Score (*F*1)	$F1=2\cdot \frac{Precision\;\cdot \;Recall}{Precision+Recall}=\frac{2\;\cdot \;TP}{2\;\cdot \;TP+FP+FN}\;$

## Data Availability

The original contributions presented in this study are included in the article. Further inquiries can be directed to the corresponding author.
